# Choledochotomy for Obstructive Choledocholithiasis in Two Dogs

**DOI:** 10.1155/2019/4748194

**Published:** 2019-07-24

**Authors:** Christian Folk, Cassie Lux

**Affiliations:** The University of Tennessee College of Veterinary Medicine, Knoxville, Tennessee, USA

## Abstract

Two geriatric (> 9 years old) dogs presented for vomiting, lethargy, and anorexia. Obstructive choledocholithiasis was diagnosed based on analysis of a serum biochemical analysis and abdominal radiographs and ultrasonography. In both dogs, choledocholiths were removed from the common bile duct via a choledochotomy without a cholecystectomy, and survival without recurrence occurred in both dogs for more than 1 year.

## 1. Introduction

Choledocholithiasis is an uncommon disease process in dogs, which can result in life-threatening extrahepatic biliary obstruction. Cholecystectomy has previously been considered the treatment of choice for obstructive cholelithiasis when the choleliths are predominantly within the gallbladder. When cholelithiasis is present throughout the biliary tree, it is less obvious which surgical treatment is ideal to alleviate the biliary obstruction [1–8]. The cases presented here describe choledochotomy and biliary stent placement alone for treatment of extrahepatic biliary obstruction, which has previously been unreported.

## 2. Case 1

A nine-year-old 7-kg (15.4-lb) spayed female mixed breed dog (dog one) was examined for a 4 day history of vomiting, lethargy, and anorexia. On initial physical examination, a grade III/VI systolic heart murmur was detected with maximum intensity over the mitral valve region. A complete blood count (CBC) revealed unremarkable findings. A serum biochemical analysis revealed a normal total bilirubin (0.5 mg/dL; reference range 0.1-0.6 mg/dL) and elevations in alkaline phosphate (ALP) (435 U/L; reference range 13-240 U/L), alanine transferase (ALT) (1259 U/L; reference range 18-100 U/L) and cholesterol (375 mg/dL; reference range 130-354 mg/dL).

**Figure 1:**
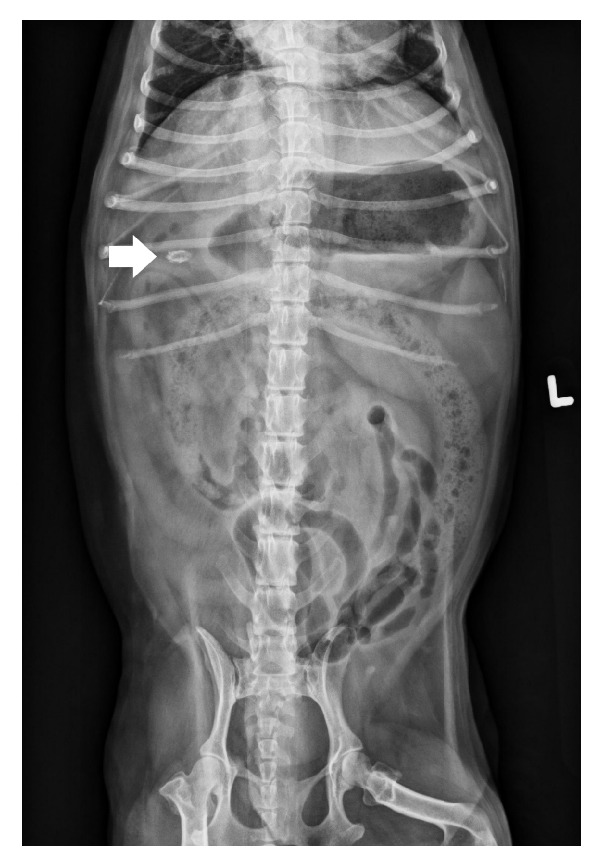
Ventrodorsal radiograph of the abdomen. Note the oval-shaped radiopaque calculi in the right cranial abdomen (white arrow).

The patient underwent standard three-view abdominal radiography, which revealed a 1.1 cm x 0.6 cm oval-shaped mineral opacity in the right cranial abdomen superimposed with the proximal descending duodenum (Figure 1). Transabdominal ultrasonography was also performed, and findings included multiple hyperechoic structures with acoustic shadowing within the common bile duct (CBD) at the distal-most aspect adjacent to the major duodenal papilla with the largest one measuring approximately 1 cm in diameter. The CBD was found to be markedly distended along its entire length, measuring up to 8 mm in diameter. The average diameter of the normal canine CBD is 3 mm [9]. The gallbladder (GB) was markedly distended and the wall appeared thick and irregular. The canine GB wall typically measures 2-3 mm in thickness and wall thickening of the GB is diagnosed when the wall is thicker than 3-3.5 mm [10]. The GB lumen contained a large amount of variably echogenic debris. The majority of the debris was non-organized and collected in the dependent portion while some appeared adhered to the wall and was partially organized suggestive of early mucocele formation.

Based on clinical signs, clinicopathologic findings, and results of diagnostic imaging, the primary differential diagnosis was partial obstruction of the extrahepatic biliary tract due to choledocholithiasis [1, 2]. The dog's clinical signs improved with supportive care of intravenous fluid therapy, maropitant (1 mg/kg, IV, q 24 hr), and buprenorphine (0.02 mg/kg, IV, q 8 hr), and an abdominal ultrasound was performed the following day revealing the common bile duct to be slightly less distended measuring 4-5 mm in diameter with the previously described choledocholiths in a similar location. The dog was discharged with medical management including ursodeoxycholic acid (7.7 mg/kg, PO, q 12 hr), amoxicillin-clavulanic acid (16 mg/kg, PO, q 12 hr), tramadol (6.5 mg/kg, PO, q 8-12 hours) and s-adenosylmethionine and silybin (30 mg/kg, PO, q 24 hr).

The dog represented to the emergency service 3 days later for icterus, anorexia, and lethargy. On physical examination, the dog now exhibited generalized icterus and marked discomfort was elicited on deep palpation of the abdomen. A serum chemical analysis revealed elevations in ALP (903 U/L; reference range 13-240 U/L), ALT (1250 U/L; reference range 18-100 U/L), aspartate aminotransferase (AST) (80 U/L; reference range 9-63 U/L), cholesterol (557 mg/dL; reference range 130-354 mg/dL), and total bilirubin (7.6 mg/dL; reference range 0.1-0.6 mg/dL).

**Figure 2:**
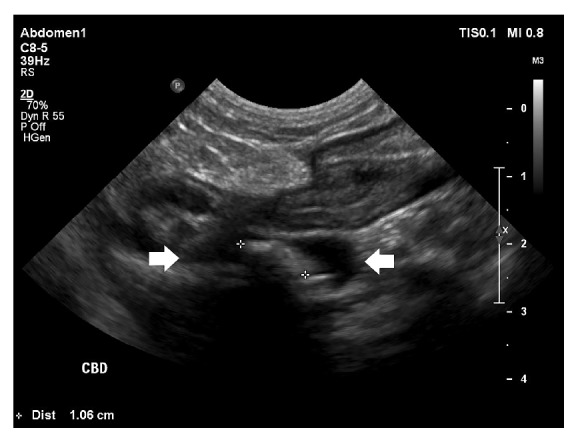
Abdominal ultrasonography. Note the 1.06 cm choledocholith (white crosshairs) obstructing the entrance of the common bile duct into the duodenum resulting in marked bile duct distension (white arrows).

Transabdominal ultrasonography revealed the GB and CBD to be markedly distended with the CBD measuring up to 8 mm in diameter. Several choledocholiths were noted within the lumen of the cystic duct and CBD (Figure 2). The wall of the distal CBD was markedly thickened and hyperechoic, measuring up to 3 mm in thickness. The wall of the canine CBD is often partially visualized with ultrasonography and should not exceed 1 mm in thickness [11]. In addition, mild dilation of several intrahepatic bile ducts with intraluminal choleliths was noted. The variably echogenic, partially organized, and adhered debris within the GB lumen was unchanged compared to the transabdominal ultrasound performed 3 days prior. The decision was made to perform an immediate exploratory laparotomy due to the suspicion of complete extrahepatic biliary tract obstruction from choledocholithiasis. Echocardiography demonstrated mild mitral and tricuspid regurgitation with normal chamber sizes and a normal left ventricular systolic function. A coagulation panel consisting of prothrombin time (PT) and partial prothrombin time (PTT) was done prior to surgery and both values were within normal reference ranges.

General anesthesia was induced with administration of fentanyl (5.0 mcg/kg, IV), ketamine (2 mg/kg, IV), and propofol (4 mg/kg, IV) to effect. An endotracheal tube was placed, and anesthesia was maintained with delivery of sevoflurane in oxygen, with a constant rate infusion (CRI) of fentanyl (3 to 5 mcg/kg/hr, IV) and lidocaine (25 to 50 mcg/kg/min, IV) for analgesia. After standard aseptic preparation for surgery, and with the patient positioned in dorsal recumbency, a ventral midline incision was made for exploratory celiotomy. An enlarged liver, a moderately distended GB, and markedly dilated CBD were noted during abdominal exploration.

Multiple stones were palpated within the lumen of the common bile duct. An antimesenteric duodenal incision measuring 2 cm in length was made with a #11 blade distal to the externally visible junction of the CBD and duodenum, and the major duodenal papilla was visualized. The largest choledocholith was isolated within the lumen of the common bile duct, and a #11 blade was used to make a 1 cm incision into the CBD over the choledocholith. The 1 cm in length choledocholith was removed and the CBD was gently compressed dislodging multiple stones for evacuation. A five French red rubber catheter (Covidien Dover Rob-Nel, Medtronic Covidien, Minneapolis, MN 55432) was placed through the major duodenal papilla, bypassing the choledochotomy, and the cystic and intrahepatic bile ducts were flushed copiously with sterile saline. This process was repeated multiple times alongside massaging the GB, cystic ducts, and intrahepatic bile ducts to dislodge and remove any remaining biliary sludge and stones. The CBD incision was closed with a single layer closure using monofilament absorbable suture (size 4-0 polydioxanone (PDS II, Ethicon, Johnson and Johnson, Somerville, NJ 08876)) in a simple continuous pattern. The red rubber catheter was then sutured to the lumen of the duodenum with a simple interrupted pattern of monofilament absorbable suture (size 3-0 polydioxanone (PDS II, Ethicon, Johnson and Johnson, Somerville, NJ 08876)) to act as a stent. The duodenal incision was closed with a modified Gambee pattern using monofilament absorbable suture (size 4-0 polydioxanone (PDS II, Ethicon, Johnson and Johnson, Somerville, NJ 08876)). A guillotine biopsy of the left medial liver lobe was collected and submitted for histopathologic examination. Choleliths and liver tissue were submitted for aerobic and anaerobic culture. Several of the choledocholiths were submitted for quantitative analysis. The abdomen was then copiously lavaged with sterile saline and then closed routinely. Postoperatively, the dog was treated with ampicillin (30 mg/kg, IV, q 8 hr) for 3 days for suspected cholecystitis. Pain was managed postoperatively with a fentanyl CRI (2-5 mcg/kg/hr, IV) that was tapered over 72 hours of hospitalization.

Histopathological examination of the liver revealed moderate multifocal hepatic glycogenosis with biliary hyperplasia. No organisms were detected with aerobic and anaerobic cultures of the liver and gallbladder. Composite analysis of the collected gallstones revealed the largest calculus to have a surface layer composed of 100% calcium carbonate with a homogeneous interior composed of 100% bilirubin.

Four days postoperative, a serum biochemical analysis revealed persistent but improved elevations in ALP (440 U/L; reference range 13-240 U/L) and ALT (330 U/L; reference range 18-100 U/L). The dog's hypercholesteremia, hyperbilirubinemia, and elevated AST had resolved, but the albumin had decreased (1.6 g/dL; reference range 3.2-4.3 g/dL). The dog was transitioned from intravenous to oral analgesics which included acetaminophen (10 mg/kg, PO, q 8 hr) and codeine (1.5 mg/kg, PO, q 8 hr). The clinical signs at presentation had resolved, the dog was eating and subsequently was discharged on day 4 postoperatively. Follow up information was obtained from a phone conversation with the dog's owners. The owners did not report any return of clinical signs related to biliary obstruction in the 16 months since surgery.

## 3. Case 2

A thirteen-year-old 5.0-kg (11-lb) spayed female Shih Tzu (dog two) was referred for evaluation of a 7 day history of vomiting, lethargy, and anorexia. On initial examination, the dog's sclera were icteric and marked discomfort was elicited on deep palpation of the abdomen. A CBC revealed unremarkable findings. Alkaline phosphate (3788 U/L; reference range 13-240 U/L), ALT (3851 U/L; reference range 18-100 U/L), AST (247 U/L; reference range 9-63 U/L), total bilirubin (4.3 mg/dL; reference range 0.1-0.6 mg/dL), gamma-glutamyltransferase (GGT) (81 mg/dL; reference range 0-5 mg/dL), and cholesterol (499 mg/dL; reference range 130-354 mg/dL) were elevated.

Abdominal radiography was performed revealing numerous, pinpoint, round mineral opaque foci within the right cranioventral aspect of the liver, and four approximately 0.5 cm in diameter mineral opaque structures were noted within the mid cranial abdomen superimposed with the gastric body and liver. Transabdominal ultrasonography revealed several intrahepatic ducts to be markedly distended with multiple small to moderately sized hyperechoic structures up to 0.5 cm in diameter. The gallbladder was markedly distended with a thickened and irregular wall. The CBD was markedly dilated (around 1 cm in diameter) with thickened and irregular walls and contained echogenic sediment and multiple hyperechoic structures consistent with choledocholiths. The largest choledocholith was just proximal to the major duodenal papilla.

The decision was made to perform an exploratory celiotomy because of presumptive complete extrahepatic biliary obstruction due to choledocholithiasis. A coagulation panel consisting of PT and PTT was done prior to surgery revealing values within normal reference ranges. General anesthesia was induced with administration of butorphanol (0.3 mg/kg, IV), dexmedetomidine (2 mcg/kg, IV), ketamine (2 mg/kg, IV), and propofol (4 mg/kg, IV) to effect. Anesthesia was maintained with delivery of sevoflurane in oxygen, with a CRI of fentanyl (3 to 5 mcg/kg/hr, IV) and a CRI of lidocaine (25-50 mcg/kg/min, IV).

An exploratory celiotomy was performed via a ventral midline incision. A markedly dilated common bile duct was noted and a #11 blade was used to make a 1 cm long choledochotomy. Multiple small, green, spiculated choledocholiths were expelled along with a 1 cm in length choledocholith. The CBD was gently compressed starting distally and moving proximally which dislodged multiple stones for removal. A duodenotomy was performed with a #11 blade on the antimesenteric border to visualize the major duodenal papilla. A five French red rubber catheter (Covidien Dover Rob-Nel, Medtronic Covidien, Minneapolis, MN 55432) was placed through the papilla bypassing the choledochotomy, and the bile ducts were flushed copiously with sterile saline while massaging the biliary tree to dislodge and remove any remaining biliary sludge and stones within the intrahepatic ducts, gallbladder, and CBD. The red rubber catheter (Covidien Dover Rob-Nel, Medtronic Covidien, Minneapolis, MN 55432) was sutured to the lumen of the duodenum with monofilament absorbable suture (4-0 polydioxanone (PDS II, Ethicon, Johnson and Johnson, Somerville, NJ 08876)) in a simple interrupted pattern to perform as a stent. The choledochotomy was closed with a simple continuous pattern using monofilament absorbable suture (4-0 polydioxanone (PDS II, Ethicon, Johnson and Johnson, Somerville, NJ 08876)). The duodenal incision was closed with a simple interrupted pattern using monofilament absorbable suture (4-0 polydioxanone (PDS II, Ethicon, Johnson and Johnson, Somerville, NJ 08876)). A guillotine biopsy of the left medial liver lobe was collected and submitted for histopathologic examination. Tissue samples of the liver were collected aseptically and submitted for culture. The abdomen was then copiously lavaged with sterile saline and closed routinely. Pain was managed postoperatively with a fentanyl CRI (2-5 mcg/kg/hr, IV) that was tapered over a 24-hour period and transitioned to buprenorphine (0.03 mg/kg, IV, q 4-6 hr) for the remainder of hospitalization.

Histopathological examination of the liver revealed findings suggestive of microvascular dysplasia. No organisms were detected with aerobic and anaerobic cultures of the liver.

Collected gallstones were not submitted for composite analysis.

One day postoperative, a serum biochemical analysis revealed persistent but improved elevations in ALP (1433 U/L; reference range 13-240 U/L), GGT (27 U/L; reference range 0-5 U/L), and ALT (897 U/L; reference range 18-100 U/L). The dog's hypercholesteremia, hyperbilirubinemia, and elevated AST had resolved, though the albumin had decreased (1.7 g/dL; reference range 3.2-4.3 g/dL). The dog was transitioned from intravenous to oral analgesics which included tramadol (5 mg/kg, PO, q 8 hr) and gabapentin (10 mg/kg, PO, q 8 hr). The clinical signs at presentation had resolved, the dog was eating and subsequently was discharged on day 3 postoperatively. Follow up information was obtained from a phone conversation with the dog's owners. The owners did not report any return of clinical signs related to biliary obstruction in the 14 months since surgery.

## 4. Discussion

Cholelithiasis in dogs is an uncommon cause of a clinically severe disease, where most affected dogs do not exhibit clinical signs [1–8]. Medical dissolution generally involves ursodeoxycholic acid and broad-spectrum antibiotics but in most cases is not successful. Clinical signs associated with obstructive cholelithiasis are usually non-specific and include anorexia, vomiting, diarrhea, lethargy, icterus, and abdominal pain and distension as seen in both dogs [6, 7]. Biochemical and hematological abnormalities are indicative of biliary obstruction, but not specific to cholelithiasis. In the case of complete extrahepatic bile duct obstruction (EHBDO) due to cholelithiasis, emergency surgery is warranted.

Extrahepatic biliary tract disease is historically associated with relatively high morbidity and mortality in dogs with mortality rates reported between 22 to 64% [12–16]. Cholecystectomy is considered the treatment of choice for obstructive choledocholithiasis if the choledocholiths can be massaged into the GB and the CBD is patent. Other surgical options include cholecystotomy and biliary diversion procedures such as cholecystoenterostomies to prevent bile passage through a damaged common bile duct. It has been found that dogs that survive the early postoperative period following biliary surgery generally have a good long-term prognosis unless they have undergone a biliary diversion procedure [12, 17].

In the cases reported here, it was not attempted to massage the distal, largest choledocholiths proximally back into the GB due to worry of damaging and/or rupturing an already inflamed CBD. To prevent iatrogenic trauma from attempting to massage the distal stones proximally, choledochotomies were performed directly over the distal, largest choledocholiths. Once the largest choledocholiths were removed, all other palpable choleliths were able to be removed at this site from the GB, cystic duct, and CBD with gentle manipulation and copious lavage with saline. Given the inability to remove all of the choleliths within the intrahepatic biliary ducts, it was elected to perform a choledochotomy without a cholecystectomy in both dogs. This would allow the gallbladder to be available for future procedures should the remaining intrahepatic choleliths migrate and cause recurrent extrahepatic biliary obstruction. Choleliths can initiate within the gallbladder or the biliary ducts due to inflammation, infection, or biliary stasis, and further biliary stasis from partial or complete biliary obstruction and potentiate new cholelith formation throughout the biliary tree [1, 2, 5, 6]. Therefore, it is difficult to determine if the GB is a true source of disease in dogs with such extensive cholelithiasis. Neither dog had bacterial growth on aerobic or anaerobic bacterial cultures of the GB and liver, nor did they have evidence of inflammation on hepatic biopsy to aid in the identification of the initiating factor for cholelithiasis. It is a valid consideration to suspect that due to the chronicity involved in cholelith formation that chronic CBD inflammation and passage or intermittent movement of smaller choleliths had been occurring for some time prior to acute obstruction in the cases described.

Some reported complications following choledochotomy include postoperative bile leakage, dehiscence, stricture, and adhesion formation [16, 18]. Risks factors for complications following choledochotomy have not been proven but are theorized to include increased tension on the repair, tissue friability and the presence of infection [14]. Most choledochotomies are performed in conjunction with cholecystectomy for the treatment of EHBDO and cholelithiasis.

Choledochotomy alone has historically been shown to be associated with high mortality (100%) and poor long-term prognosis (< 6 months) [6]. One study has shown that choledochotomy performed with cholecystectomy was associated with low perioperative morbidity and no mortality in a small cohort of cases [18].

To the authors' knowledge, there are no reported cases of dogs undergoing choledochotomy without cholecystectomy for obstructive choledocholithiasis that exhibited low morbidity and a long-term prognosis of greater than 6 months. This case report illustrates that a choledochotomy without cholecystectomy is a viable surgical treatment option for obstructive choledocholithiasis when choleliths are present throughout the biliary tree and removal of the gallbladder is not possible. Performing choledochotomy alone can minimize the known risks and complications associated with performing multiple extrahepatic biliary tract procedures, and allow for biliary rerouting procedures involving the GB with recurrent biliary obstruction due to choleliths.

## Abbreviations

ALP: Alkaline phosphate
ALT: Alanine transferase
AST: Aspartate aminotransferase
CBC: Complete blood count
CBD: Common bile duct
CRI: Constant rate infusion
EHBDO: Extrahepatic bile duct obstruction
GB: Gallbladder
GGT: Gamma-glutamyltransferase
PT: Prothrombin time
PTT: Partial prothrombin time.

## References

[1] Fahie M., Martin R. (1995). Extrahepatic biliary tract obstruction: a retrospective study of 45 cases (1983-1993). *Journal of the American Animal Hospital Association*.

[2] Neer T. M. (1992). A review of disorders of the gallbladder and extrahepatic biliary tract in the dog and cat. *Journal of Veterinary Internal Medicine*.

[3] Matthiesen D. T., Lammerding J. (1984). Gallbladder rupture and bile peritonitis secondary to cholelithiasis and cholecystitis in a dog.. *Journal of the American Veterinary Medical Association*.

[4] Harris S. J., Simpson J. W., Thoday K. L. (1984). Obstructive cholelithiasis and gall bladder rupture in a dog. *Journal of Small Animal Practice*.

[5] Schall W. D., Chapman W. L., Finco D. R. (1973). Cholelithiasis in dogs. *Journal of the American Veterinary Medical Association*.

[6] Kirpensteijn J., Fingland R. B., Ulrich T. (1993). Cholelithiasis in dogs: 29 cases (1980-1990). *Journal of the American Veterinary Medical Association*.

[7] Guilford W. G., Center S. A., Strombeck D. R. (1990). *Small Animal Gastroenterology*.

[8] Kanemoto H., Fukushima K., Tsujimoto H., Ohno K. (2017). Intrahepatic cholelithiasis in dogs and cats: a case series. *Canadian Veterinary Journal*.

[9] Long C. (2004). Hepatobiliary ultrasound. *American College of Veterinary Internal Medicine*.

[10] Spaulding K. A. (1993). Ultrasound corner gallbladder wall thickness. *Veterinary Radiology & Ultrasound*.

[11] Dominique P., Marc-André D. (2015). *Atlas of Small Animal Ultrasonography*.

[12] Amsellem P. M., Seim H. B., MacPhail C. M. (2006). Long-term survival and risk factors associated with biliary surgery in dogs: 34 cases (1994–2004). *Journal of the American Veterinary Medical Association*.

[13] Pike F. S., Berg J., King N. W., Penninck D. G., Webster C. R. (2004). Gallbladder mucocele in dogs: 30 cases (2000–2002). *Journal of the American Veterinary Medical Association*.

[14] Mehler S. J., Mayhew P. D., Drobatz K. J., Holt D. E. (2004). Variables associated with outcome in dogs undergoing extrahepatic biliary surgery: 60 cases (1988-2002). *Veterinary Surgery*.

[15] Mayhew P. D., Holt D. E., McLear R. C., Washabau R. J. (2002). Pathogenesis and outcome of extrahepatic biliary obstruction in cats. *Journal of Small Animal Practice*.

[16] Matthiesen D. T. (1989). Complications associated with surgery of the extrahepatic biliary system.. *Problems in Veterinary Medicine*.

[17] Mayhew P. D., Richardson R. W., Mehler S. J., Holt D. E., Weisse C. W. (2006). Choledochal tube stenting for decompression of the extrahepatic portion of the biliary tract in dogs: 13 cases (2002–2005). *Journal of the American Veterinary Medical Association*.

[18] Baker S. G., Mayhew P. D., Mehler S. J. (2011). Choledochotomy and primary repair of extrahepatic biliary duct rupture in seven dogs and two cats. *Journal of Small Animal Practice*.

